# Draft Genome Sequence of *Mycobacterium acapulcensis* Strain CSURP1424

**DOI:** 10.1128/genomeA.00836-16

**Published:** 2016-08-11

**Authors:** Shady Asmar, Nicolás Rascovan, Catherine Robert, Michel Drancourt

**Affiliations:** Unité de Recherche sur les Maladies Infectieuses et Tropicales Émergentes, CNRS, UMR 7278, IRD 198, Faculté de Médecine, Aix-Marseille Université, Marseille, France

## Abstract

*Mycobacterium acapulcensis* is a rapidly growing scotochromogenic acid-fast bacillus. The draft genome of *M. acapulcensis* CSURP1424 comprises 5,290,974 bp, exhibiting a 66.67% G+C content, 4,870 protein-coding genes, and 71 predicted RNA genes.

## GENOME ANNOUNCEMENT

*Mycobacterium acapulcensis* is a rapidly growing scotochromogenic acid-fast bacillus that was first isolated from sputum in Acapulco, a town on the Pacific coast of Mexico, during a campaign against tuberculosis (1). *M. acapulcensis* was regarded for a long time as a synonym of *Mycobacterium flavescens*, a closely related species (2, 3).

We performed whole-genome sequencing of *M. acapulcensis* CSURP1424 in order to facilitate the development of advanced molecular tools for the detection and identification of this species.

Genomic DNA was isolated from *M. acapulcensis* CSURP1424 cultured in MGIT Middlebrook liquid culture (Becton, Dickinson, Le Pont-de-Claix, France) at 37°C in a 5% CO_2_ atmosphere. *M. acapulcensis* genomic DNA was sequenced by Illumina MiSeq runs (Illumina Inc, San Diego, CA, USA). A library with a 5.48-kb insert was loaded twice on a flow cell. Within these runs, the index representation for *M. acapulcensis* was determined to be 7.33% and 5.99%. The 1,841,967 paired reads were trimmed using Trimmomatic (4) and then assembled into scaffolds using Spades version 3.5 (5, 6) before finishing. Contigs were combined together by SSPACE version 2 (7) and Opera version 2 (8) helped by GapFiller version 1.10 (9). This resulted in a draft genome consisting of 29 scaffolds and 97 contigs, for a total of 5,290,974 bp and a G+C content of 66.67%. Noncoding genes and miscellaneous features were predicted using RNAmmer (10), ARAGORN (11), Rfam (12), PFAM (13), and Infernal (14). Coding DNA sequences (CDSs) were predicted using Prodigal (15), and functional annotation was achieved using BLASTp against the GenBank database (16) and the Clusters of Orthologous Groups (COGs) database (17, 18). The genome was shown to encode at least 71 predicted RNAs, including four rRNAs, 46 tRNAs, one tmRNAs, and 20 miscellaneous RNAs. A total of 4,920 identified genes yielded a coding capacity of 4,563,841 bp (coding percentage, 86.25%). Among these genes, 3,915 (80.39%) were found to encode for putative proteins and 723 (14.85%) were assigned as hypothetical proteins. Moreover, 2,929 (60.1%) genes matched at least one sequence in the Clusters of Orthologous Groups database with BLASTp default parameters. Further, the *M. acapulcensis* CSURP1424 genome was incorporated into *in silico* DNA-DNA hybridization (DDH) (19) with reference genomes selected on the basis of their 16S rRNA gene proximity, and DDH values were estimated using the GGDC version 2.0 online tool (20). This analysis yielded 22.8% ± 2.37 similarity with *Mycobacterium pyrenivorans* (21); 21.3% ± 2.34 with *Mycobacterium rhodesiae* NBB3 (22) and *Mycobacterium tusciae* (23); 21.1% ± 2.33 with *Mycobacterium austroafricanum* (24) and *Mycobacterium vanbaalenii* (25); 20.8% ± 2.33 with *Mycobacterium gilvum* Spyr1 (26); 20.5% ± 2.32 with *Mycobacterium aurum* (27); 20.3% ± 2.31 with *Mycobacterium fortuitum* ATCC 6841 (28); 20% ± 2.31 with *Mycobacterium neoaurum* DSM 44074 (29); and 19.9% ± 2.3 with *Mycobacterium marinum* E11 (30).

### Accession number(s).

The *M. acapulcensis* genome sequence has been deposited at EMBL under the accession numbers LT592221 to LT592249.
